# Invasive *Fusarium solani* infection diagnosed by traditional microbial detection methods and metagenomic next-generation sequencing in a pediatric patient: a case report and literature review

**DOI:** 10.3389/fmed.2024.1322700

**Published:** 2024-07-08

**Authors:** Jiaji Ling, Liting Liang, Xingxin Liu, Wenjing Wu, Ziyi Yan, Wei Zhou, Yongmei Jiang, Linghan Kuang

**Affiliations:** ^1^Department of Laboratory Medicine, West China Second University Hospital, Sichuan University, Chengdu, China; ^2^Key Laboratory of Birth Defects and Related Diseases of Women and Children (Sichuan University), Ministry of Education, Chengdu, China

**Keywords:** *Fusarium solani*, invasive fungal infections, detection, MALDI-TOF MS, Sanger sequencing, mNGS

## Abstract

*Fusarium solani*, as an opportunistic pathogen, can infect individuals with immunosuppression, neutropenia, hematopoietic stem cell transplantation (HSCT), or other high-risk factors, leading to invasive or localized infections. Particularly in patients following allogeneic HSCT, *Fusarium solani* is more likely to cause invasive or disseminated infections. This study focuses on a pediatric patient who underwent HSCT for severe aplastic anemia. Although initial blood cultures were negative, an abnormality was detected in the 1,3-β-D-glucan test (G test) post-transplantation. To determine the causative agent, blood samples were subjected to metagenomic next-generation sequencing (mNGS) and blood cultures simultaneously. Surprisingly, the results of matrix-assisted laser desorption/ionization time-of-flight mass spectrometry (MALDI-TOF MS) and mNGS differed slightly, with mNGS identifying *Nectria haematonectria*, while MALDI-TOF MS based on culture showed *Fusarium solani*. To clarify the results, Sanger sequencing was performed for further detection, and the results were consistent with those of MALDI-TOF MS. Since the accuracy of Sanger sequencing is higher than that of mNGS, the diagnosis was revised to invasive *Fusarium solani* infection. With advancements in technology, various detection methods for invasive fungi have been developed in recent years, such as mNGS, which has high sensitivity. While traditional methods may be time-consuming, they are important due to their high specificity. Therefore, in clinical practice, it is essential to utilize both traditional and novel detection methods in a complementary manner to enhance the diagnosis of invasive fungal infections.

## Introduction

*Fusarium* is a common saprophytic fungus in soil and is a conditional pathogen capable of causing invasive or localized infections (18). As an opportunistic pathogen, *Fusarium* has been increasingly reported to cause invasive or localized infections in humans in recent years, primarily reported in the United States, France, Italy, and others. However, there are limited relevant reports in China (22). In patients with normal immune function, *Fusarium* often causes superficial or localized lesions, such as onychomycosis and keratitis (12). A favorable prognosis can be achieved after active antifungal treatment in most cases (9). However, in patients undergoing organ transplantation, hematological malignancies, or allogeneic HSCT, *Fusarium* can easily cause invasive or even disseminated infections, typically affecting the lungs, blood vessels, and sinuses, as well as manifest as cranial-cerebral infections (20).

As an opportunistic pathogen, *Fusarium* infection is not as common as *Aspergillus* infection. Some *Fusarium* species are opportunistic pathogens that mainly cause local infections in individuals with normal immune systems (10). However, *Fusarium* can lead to invasive infections in patients with malignant hematological diseases, aplastic anemia, organ transplantation, or those undergoing chemotherapy (18, 20). Disseminated *Fusarium* disease almost only occurs in immunocompromised individuals, especially in cancer patients with neutropenia caused by cytotoxic drug therapy or bone marrow transplantation (13, 21). Granulocytes and macrophages play an important role in the immune defense against *Fusarium* (33). *Fusarium* can cause disseminated infections in patients with severely compromised immunity. In some research centers, *Fusarium* is the second most common pathogen after *Aspergillus* infection in high-risk populations such as leukemia patients, solid organ transplant recipients, and allogeneic bone marrow or stem cell transplant recipients (1). The prognosis of disseminated *Fusarium* disease, which can be life-threatening, is largely influenced by immune status (13, 21, 30).

*Fusarium* mainly works by producing *Fusarium* toxins; some of them produce A-type and B-type trichothecene mycotoxins (25). The A-type trichothecene includes various toxins such as T-2 toxin and HT-2 toxin. Of these, T-2 toxin is the most important toxin in this group because it inhibits eukaryotic protein synthesis and is highly toxic to white blood cells, often leading to immune suppression. B-type trichothecene mycotoxins include nivalenol, deoxynivalenol, 3-/15 acetyl nivalenol, and Fusarenon-X. Scientific research has shown that these toxins affect the immune system of experimental animals, leading to their increased susceptibility to various pathogens (7, 16).

In the past, invasive fungal infections were mainly diagnosed through host factors, clinical features, microbiological examination, and histopathology. With the rapid progress of society, novel technologies for diagnosing invasive fungal infections have emerged, such as polymerase chain reaction-based methods and metagenomic next-generation sequencing. In this study, we diagnosed an invasive *Fusarium solani* infection with the collaboration of traditional microbial detection methods and metagenomic next-generation sequencing in a pediatric patient.

## Case presentation

A 3-year-old girl was diagnosed with severe aplastic anemia in another hospital 9 months ago. After admission to the Pediatric Hematology Department of our hospital, HSCT was scheduled according to an individualized treatment plan. Before transplantation, a chemotherapy pretreatment regimen was administered according to standard protocol. Cefoperazone sodium and tazobactam sodium were used to prevent bacterial infections, micafungin was used to prevent fungal infections, ganciclovir was used to prevent viral infections, and the combination of propranolol and rituximab was used to prevent graft-versus-host disease (GVHD). Twelve days after admission, the patient underwent HLA 7/12-compatible haploid allogeneic HSCT and HLA 5/10-compatible non-hematopoietic stem cell transplantation. Hematologic blood pressure increased during infusion, and no other abnormalities were observed.

Seventeen days after HSCT, the patient showed GVHD-related reactions, which gradually worsened. At the same time, liver function-related enzymes increased, and treatment was performed using plasma exchange. Then, the child developed intermittent fevers. Considering that the infection was worsening and C-reactive protein (CRP) levels were continuously elevated, imipenem and cilastatin sodium were administered to fight bacterial infections, and micafungin was continued to prevent fungal infections.

**Figure 1:**
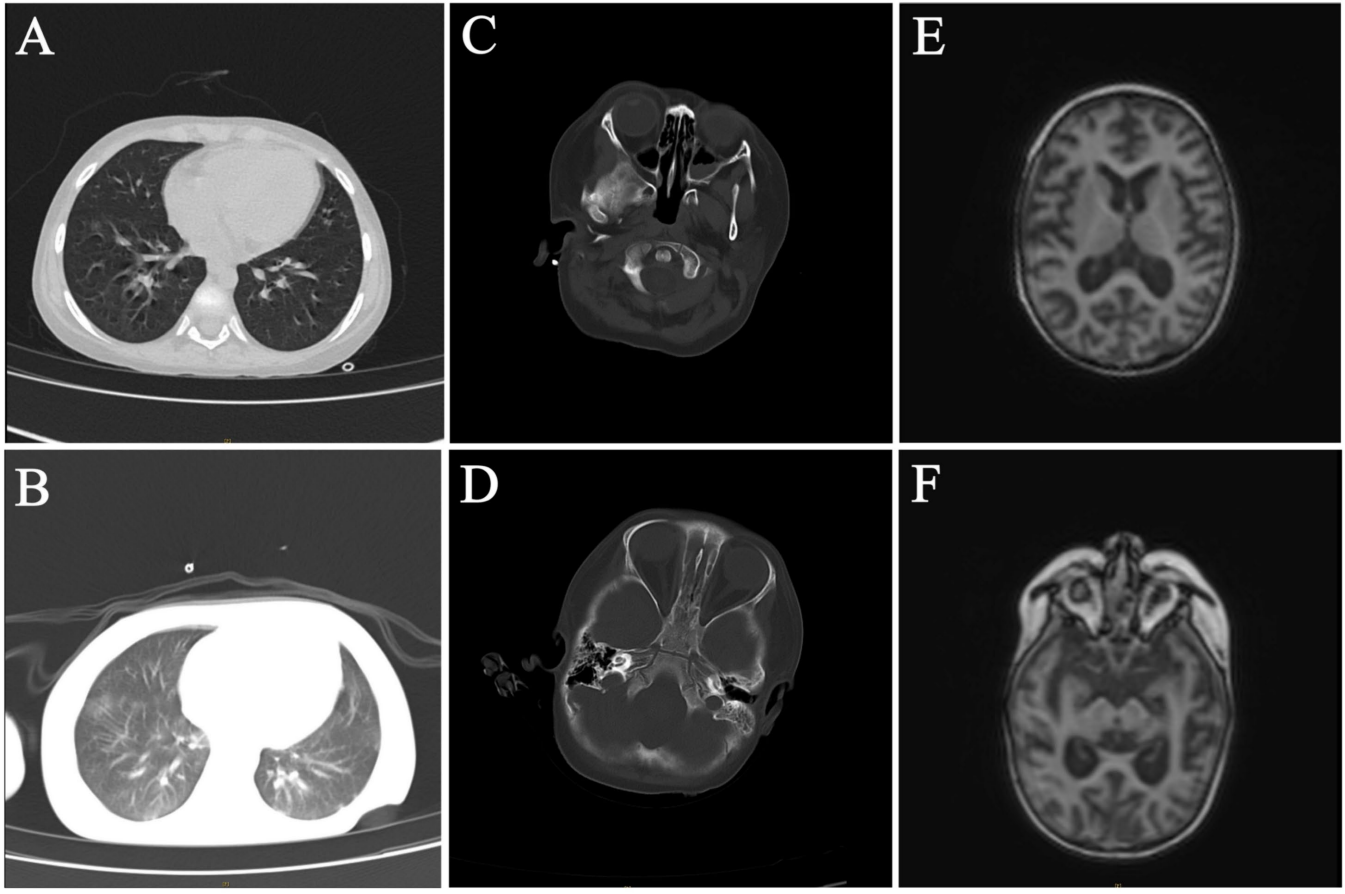
Radiological examination of the patient. **(A,B)** Chest CT on 49 days after HSCT and 81 days after HSCT. **(C–F)** Craniocerebral enhanced MRI and brain CT on 49 days after HSCT and 81 days after HSCT.

Forty-nine days after HSCT, the patient had a repeated fever with a thermal peak of 39.2°C, accompanied by increased CRP. The G test was positive at 150 pg/mL (normal value <60 pg/mL), the galactomannan test (GM test) was negative, and the blood culture was negative. A chest computed tomography (CT) examination showed multiple nodules and patchy blurred shadows in both lungs. The lower lobes of both lungs were characterized by lamellar consolidation with obvious interstitial changes (Figure 1A). Thus, voriconazole was then added to control the fungal infection.

The same treatment regimen was continued after the initial HSCT, with consecutive negative blood culture results observed during this period. Eighty-one days after HSCT, the child developed convulsions, respiratory failure with central mechanism, and electrolyte disturbance. Compared to the previous chest CT results, the lesions and interstitial changes in the lungs were aggravated (Figure 1B). Compared to 49 days after HSCT, the craniocerebral MRI with contrast and brain CT showed abnormal signals in the cortex and subcortical regions of both cerebral hemispheres, as well as in the anterior and posterior corners of both cerebral ventricles, notably in the bilateral frontal lobe and the right temporal lobe. A nodular abnormal signal was noted in the right frontal white matter area, the cerebral sulci and fissures were widened and deepened, and the bilateral lateral ventricles were widened, indicating brain atrophy changes (Figures 1C–E).

The patient’s inflammatory markers were significantly elevated (CRP 370 mg/L, PCT 1.29 ng/mL), the G test result was positive (218 pg/mL), the GM test was negative, and the blood culture remained negative. Therefore, the antifungal treatment regimen was adjusted to the combination of micafungin and amphotericin B. The patient’s condition was complex and unstable, posing a threat to life at any time. Ninety-four days after HSCT, the patient was transferred to the PICU, and the G test turned negative after treatment. However, the patient was in a coma state, and blood tests indicated significantly reduced levels of red blood cells, white blood cells, and platelets, which led to diffuse intravascular coagulation abnormalities. Continuous infusion of blood products was used to support treatment.

One hundred and sixteen days after HSCT, the G test was positive again (254 pg/mL), but the GM test was still negative. The child developed herpes lesions around her lip and body, containing bloody substances, which gradually ruptured and formed necrotic scabs. In order to identify the cause as soon as possible, the medical team performed mNGS of the pathogen in addition to the routine double-vial blood cultures. Two days later, the mNGS revealed a rare fungus, *Nectria haematonectria*. Thus, according to the guidelines, the team used voriconazole combined with micafungin antifungal therapy and abandoned the use of vancomycin and ceftazidime.

**Figure 2:**
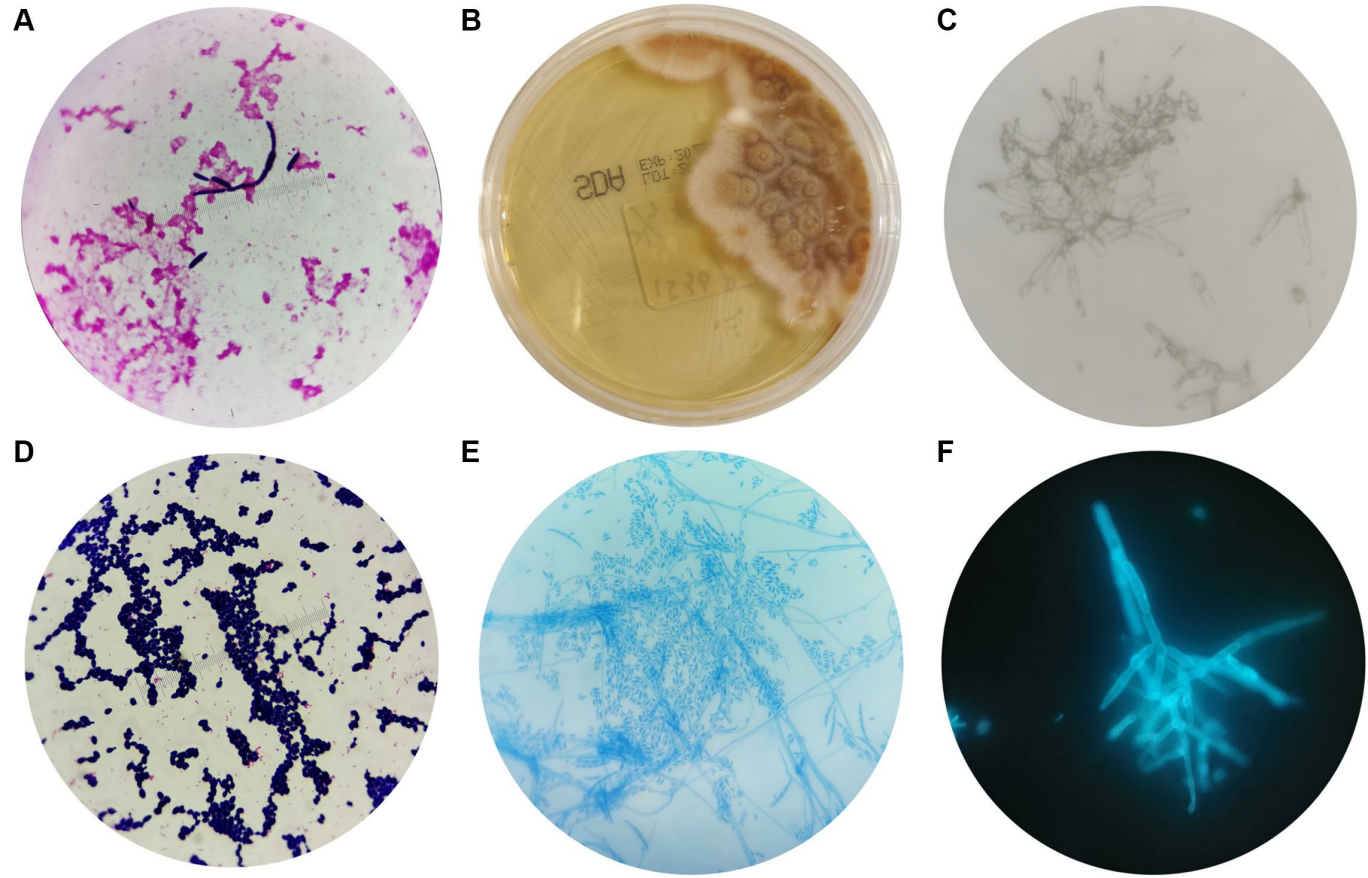
Morphological characteristics of *Fusarium solani*. **(A)** After 4 days of blood cultivation, oval fungal spores could be observed by microscope after Gram staining (magnification 10 × 100). **(B)** The morphology of *Fusarium solani* on SDA after 96 h of culture. **(C–F)** The morphologies of *Fusarium solani* were observed using a KOH fungal smear test (magnification 10 × 100), Gram staining (magnification 10 × 100), lactic acid phenol cotton blue staining (magnification 10 × 40), and fungal fluorescent staining (magnification 10 × 100).

The blood cultures showed a positive result after 4 days of culture, and oval fungal spores were observed under a microscope after Gram staining (Figure 2A), which supported our decision to initiate antifungal treatment. They were inoculated on Sabouraud agar (SDA) after 96 h of culture. The colony morphology on SDA is shown in Figure 2B; the colony exhibited dense, smooth, white mycelium, and the reverse color was reddish in the center and yellow in peripheral areas. The microstructure of the colony was observed by a KOH fungal smear test, Gram staining, lactic acid phenol cotton blue staining, and fungal fluorescent staining. Microscopic examination revealed septate, hyaline hyphae that were 4–5 microns in diameter, branching at acute angles, with numerous microconidia and sparse macroconidia. The microconidia were hyaline, unicellular to bicellular, and cylindrical to oval and formed long lateral phialides. The macroconidia were fusiform, cylindrical, moderately curved, and three or four septate (Figures 2C–F).

In addition, the colony was identified as *Fusarium solani* with 99.9% confidence using MALDI-TOF MS. However, the results did not match those obtained from mNGS. Therefore, Sanger sequencing was adopted to confirm the identity of the fungus. Prior to sequencing, genomic DNA was extracted, and agarose gel electrophoresis was performed. The Sanger sequencing results confirmed the presence of *Fusarium solani*, which was consistent with the MALDI-TOF MS findings.

To explore the minimal inhibitory concentrations (MICs) of *Fusarium solani*, an antifungal susceptibility test was performed using TDR-YEAST, and the values are summarized in Table 1. MIC values 1 and 2 represent the MICs of *Fusarium solani* isolated from aerobic and anaerobic culture bottles, respectively.

**Table 1:** Antifungal susceptibility profile of *Fusarium solani.*

Antifungal agent	MIC value 1 (μg/mL)	MIC value 2 (μg/mL)
Amphotericin B	0.03	0.03
Itraconazole	2	2
Voriconazole	0.06	0.06
Fluconazole	32	32
Flucytosine	8	8
Micafungin	0.5	0.5
Terbinafine	0.12	0.12
Caspofungin	1	1

**Figure 3:**
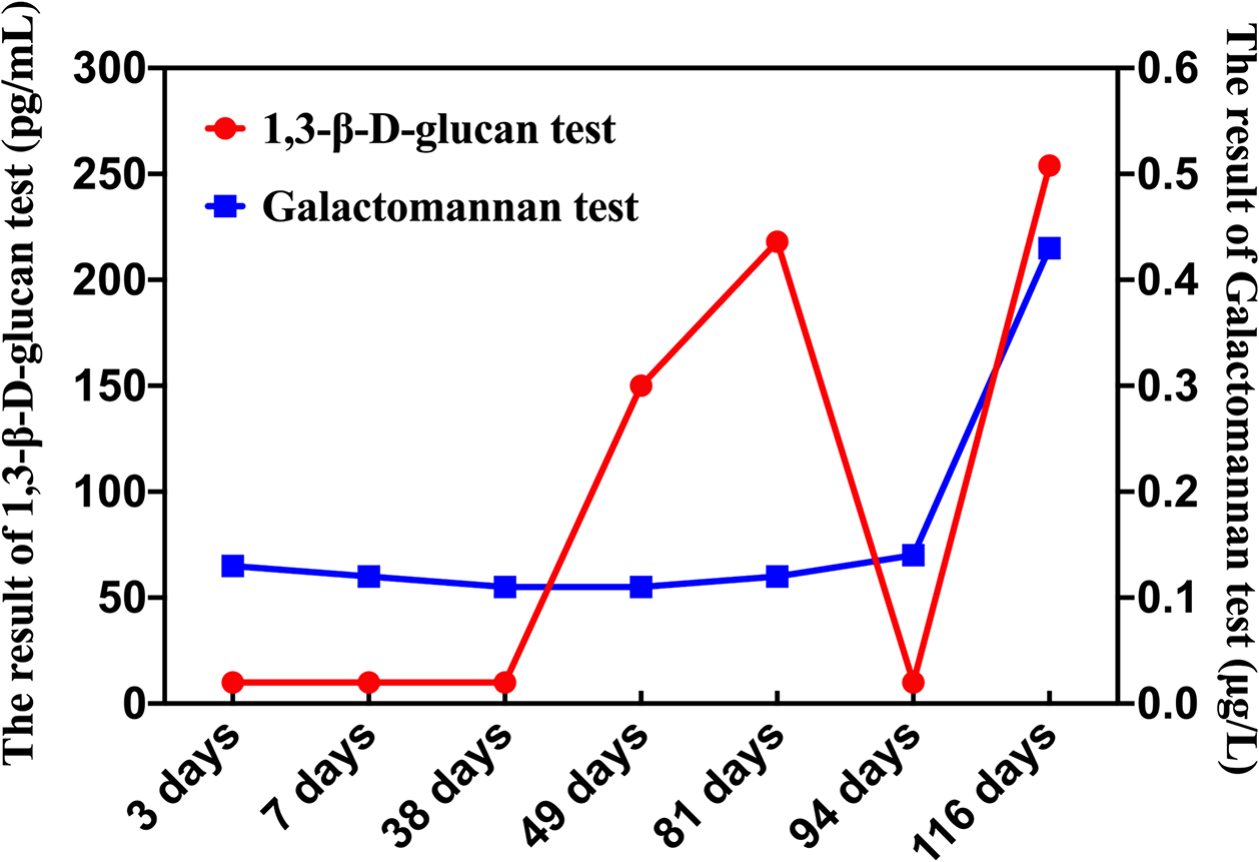
Dynamic monitoring of G and GM tests on 3, 7, 38, 49, 81, 94, and 116 days after HSCT.

Combined with multiple pieces of evidence, the patient developed recurrent fever after HSCT, indicative of a weakened immune system. Pulmonary CT scans indicated a fungal infection, with the G test showing positive results multiple times and the GM test remaining negative (Figure 3). Tests for respiratory pathogens, Epstein–Barr virus (EBV), and cytomegalovirus (CMV) were all negative. Although there were slight discrepancies between the results of MALDI-TOF MS and mNGS, Sanger sequencing was conducted for further detection and yielded consistent results with MALDI-TOF MS. Due to the higher accuracy of Sanger sequencing compared to mNGS, the diagnosis was revised to an invasive *Fusarium solani* infection. Following guidelines and MICs for *Fusarium solani*, we adjusted the anti-infective treatment regimen to include a combination of voriconazole and amphotericin B liposomes for the treatment of invasive *Fusarium solani* infection. We continued to monitor the antifungal efficacy of the treatment. Unfortunately, after more than 5 months of antifungal treatment, the child’s condition continued to deteriorate, and eventually, her parents had to face the difficult decision of discontinuing the treatment.

## Discussion

Although the efficacy of invasive *Fusarium solani* infection was not satisfactory in this study, experience can be accumulated for the management of similar cases in the future. In this study, after HSCT, successive blood cultures were negative in the early stage, but there was an abnormality in the G test. To identify the causative pathogen, blood samples were simultaneously sent for mNGS and blood culture. Interestingly, the results of MALDI-TOF MS and mNGS were different. The mNGS result was *Nectria haematonectria*, while culture-based MALDI-TOF MS showed *Fusarium solani*. To clarify the results, Sanger sequencing was performed for further detection and the results displayed the same with MALDI-TOF MS. Since the accuracy of Sanger sequencing is higher than mNGS, the diagnosis was revised to invasive *Fusarium solani* infection.

The main manifestations of invasive *Fusarium solani* infection are fever, skin appearance, and other related changes in invasive organs, but none of them are specific (31). Once infected, the skin lesions typically present as multiple erythematous papules and painful nodular lesions with central necrosis, which may appear as gangrenous purpura and can involve all parts of the body, especially the extremities. In addition, the lesions have an evolutionary stage, 75% of patients have skin lesions, and approximately 10% of patients have concentric rings. In this case, papular changes emerged at the beginning of admission, and there were cutaneous lesions of the trunk due to *Fusarium solani* infection. As we all know, invasive *Fusarium solani* infections have a poor prognosis, and patients are often accompanied by neutropenia and immunocompromised. An analysis of adult case data from the United States and Canada showed that the incidence of invasive *Fusarium* infection after HSCT was 35.4% (11). Maged et al. (17) calculated that the mortality rate of disseminated *Fusarium* infection reached 50%. A recent study of children with invasive *Fusarium* infection showed a 90-day survival rate of 77%, which is significantly higher than that of adults, but the primary disease distribution is different (2).

In this case, the results of MALDI-TOF MS and mNGS were slightly different. The mNGS result was *Nectria haematonectria*, while culture-based MALDI-TOF MS showed *Fusarium solani*. To find out the truth, Sanger sequencing was performed for further detection and the results displayed the same with MALDI-TOF MS. In order to clarify the results, many relevant studies have been read, and we have summarized the following reasons: first, Sanger sequencing has a longer read length and higher accuracy than mNGS, second, *Fusarium* is a sexual fungus of *Nectria*, so it is difficult to distinguish them using mNGS, and third, the amount of fungi in the original specimen was too small, and the background signal was too high. In addition, due to the thick fungal wall, it is difficult to break the wall to extract genomic DNA. Last but not least, improper storage of specimens led to the degradation of nucleic acids during mNGS sequencing.

## Literature review

The incidence of invasive fungal infection is on the rise with a high fatality rate (28). In order to improve the detection rate of invasive fungal infections and seize the opportunity for early treatment, many novel technologies and methods have been developed in recent years (3–6, 8, 14, 15, 19, 23, 26, 27, 29, 32, 34, 35). These novel methods are listed in Table 2 along with traditional invasive fungal detection methods.

**Table 2:** A list of traditional and novel invasive fungal detection methods.

Traditional methods	Novel methods
Microscopy	DNA sequencing
Gram staining	Polymerase chain reaction
Lactic acid phenol cotton blue staining	Sanger method
Fungal fluorescent staining	mNGS
Giemsa staining	Nucleic acid sequence-based amplification (NASBA)
Silver staining	Surface-enhanced Raman scattering (SERS)
Others	MALDI-TOF-MS-PCR
Culture-based identification	Single-cell Raman spectroscopy
Automated identification (i.e., VITEK)	Biosensors
Biochemical tests	Electrochemical impedance spectroscopy sensor
MALDI-TOF MS	Piezomicrogravimetric and electric chemosensor
Mounting medium	Piezoelectric sensor
Serology	Self-assembled gliP nanobiosensor
Antigen-detection	Label-free microcontact printing graphene-based biosensor
β-glucan	Ni/Ni(OH)_2_-rGO nanocomposite sensor
*Aspergillus galactomannan* ELISA	Oligonucleotide-capped nanoporous alumina-based sensor
	Lateral flow assay
	Fluorescent capillary electrophoresis

The treatment of disseminated *Fusarium* infection is very difficult, and the mortality rate is extremely high (24). According to the 2021 global guideline for the diagnosis and management of rare mold infections, voriconazole or a lipid formulation of amphotericin B was strongly recommended for the primary treatment of invasive fusariosis. Amphotericin B deoxycholate should not be used if other active antifungal agents are available. For other agents, a conditional recommendation is indicated. Combination therapy is frequently used in the primary treatment of invasive fusariosis because of the severity of the disease, difficulties in achieving voriconazole trough concentrations within the targeted range, and because minimum inhibitory concentrations for azoles and polyenes are often high.

Primary combination therapy, with the potential for early step-down to monotherapy later (once minimum inhibitory concentrations of azoles and polyenes are available), is an approach strongly recommended (9). In this case, despite the patient continuing to use micafungin to prevent fungal infection, they developed a *Fusarium solani* bloodstream infection, indicating resistance to echinocandin drugs. The selection of antifungal drugs should be based on the adjustment plan of antifungal susceptibility testing conducted *in vitro*. Additionally, elevated neutrophils are an important prognostic factor for this disease. The patient has had extremely low neutrophil levels since admission, with a minimum absolute neutrophil count (ANC) of 0.00 × 10^9^/L, which is also a significant factor for poor prognosis.

## Conclusion

In recent years, the incidence rate of disseminated *Fusarium* disease in patients with hematologic malignancies has increased, and the mortality rate is extremely high. Attention should be paid to the diagnosis of the disease to distinguish it from other infections and to determine the most appropriate antifungal treatment program as soon as possible. As a case study has shown, early intervention using mNGS can be beneficial, especially when conventional treatments have failed; however, it is important to carefully evaluate the accuracy of the identification results. Additionally, a combination of traditional and modern detection methods should be utilized to enhance the diagnosis of invasive fungal infections.

## Data availability statement

The datasets presented in this study can be found in online repositories. The names of the repository/repositories and accession number(s) can be found in the article/supplementary material.

## Ethics statement

The studies involving humans were approved by Medical Ethics Committee, West China Second University Hospital, Sichuan University. The studies were conducted in accordance with the local legislation and institutional requirements. The human samples used in this study were acquired from primarily isolated as part of your previous study for which ethical approval was obtained. Written informed consent for participation was not required from the participants or the participants’ legal guardians/next of kin in accordance with the national legislation and institutional requirements.

## Author contributions

JL: Conceptualization, Writing – review & editing, Methodology, Writing – original draft, Data curation, Validation. LL: Writing – review & editing, Formal analysis, Software. XL: Validation, Writing – review & editing. WW: Funding acquisition, Writing – review & editing. ZY: Funding acquisition, Writing – review & editing. WZ: Data curation, Writing – review & editing. YJ: Project administration, Resources, Writing – review & editing. LK: Funding acquisition, Resources, Supervision, Writing – review & editing.
